#  IL28B SNP genotyping among Iranian HCV-infected patients: A preliminary report

**Published:** 2011-05-01

**Authors:** Nastaran Mahboobi, Bita Behnava, Seyed Moayed Alavian

**Affiliations:** 1 Tehran Hepatitis Center, Tehran, IR Iran; 2 Baqiyatallah Research Center for Gastroenterology and Liver Diseases, Baqiyatallah University of Medical Sciences, Tehran, IR Iran

**Keywords:** IL28B, Genotype, Hepatitis C virus, Iran

Dear Editor,

At the beginning of the third millennium, Hepatitis C virus (HCV) infection continues to be a major health hazard, with 170 million chronically infected people worldwide [1]. Recent research has found that the prevalence of HCV infection in Iran is 1% and 0.1% in males and females, respectively [2]. Although a combination of pegylated interferon (PEG-IFN) and ribavirin (RBV) has been established as a standard anti-HCV therapy, treatment responses vary. Numerous factors, including gender, age, HCV RNA level prior to treatment, liver cirrhosis, and HCV genotypes have been determined to contribute to this discrepancy [1]. In addition, a number of recently published, genome-wide association studies (GWAS) have shown that polymorphisms within the IL28B locus result in differences between patients' responses to anti-HCV treatment [3][4][5][6]. In this regard, rs8099917 (8 kb upstream of the IL28B gene) and rs12979860 (3 kb upstream of the IL28B gene) have been identified as relevant single nucleotide polymorphisms (SNPs) that significantly impact treatment response to HCV infection [7]. Although both SNPs are known to be independent predictors of response to anti-HCV therapy, a recent, extensive meta-analysis has reported that rs12979860 is more closely related to treatment responses [8]. The prevalence of these SNPs and the rate of sustained viral response (SVR) have been found to vary across Caucasian, African American, and Japanese ethnicities [1]. Based on these important findings, this preliminary report presents data on IL28B SNP (rs12979860) in a group of Iranian HCV-infected patients. To our knowledge, this is the first such report published from this region, and we hope that the results will stimulate extensive research.

In this study, 68 HCV-infected patients were referred to and enrolled in 2009 at the Tehran Hepatitis Center, a primary referral hepatitis center in Iran. Of these patients, 46 (67.6%) were male and 22 (32.4%) were female, with a Mean ± SD age of 44.57 ± 13.67 years (ranging from 10 to 75 years). Whole blood (2-ml samples) in EDTA tubes was collected from these patients for genotyping analysis. DNA was extracted from 200 µl of whole blood using a pure gene Blood Core C kit (Qiagen, Hilden, Germany) according to the manufacturer's manual. The quality and the purity of extracted DNA were measured with a NanoDrop 1000 spectrophotometer. Real-time PCR was performed on IL28B SNP (rs12979860) using Roche LightCycler 480 II according to Ge, et al.'s publication [3]. The genotyping results were reported as C/C, C/T, and T/T. The SNP genotyping results for the study group, as shown in Table 1, showed C/C in 52.9% (No.= 36), C/T in 39.7% (No. = 27) and T/T in 7.3% of patients (No. = 5). Table 2 shows HCV genotypes of the study group according to IL28B SNP genotyping. The table shows that C allele, which is known as the protective allele, was present in more than 90% of the HCV-infected patients in this Iranian sample. This is in accordance with what has been reported in a previous study among healthy individuals, in which the C allele was reported to be more frequent, especially in East Asia. In that study, conducted by Thomas, et al. the authors have suggested that IL28B is under active selection, especially in the abovementioned geographic region [1]. Accordingly, our results support this hypothesis in Iran, a country located in southwestern Asia. Moreover, we believe that our findings can explain previous observations demonstrating the higher rates of SVR among Iranian HCV-infected patients in comparison with similar patients from other countries [9]. As previously described, Iranian HCV-infected patients with genotype 1 responded better than expected to anti-HCV therapy, and some hypotheses were made to justify this observation. The high prevalence of C/C and C/T genotypes of rs12979860 compared with the low prevalence of T/T among this sample of HCV-infected patients is one possible reason for the higher SVR rate. To draw a more robust conclusion in this regard, a cohort study involving a large number of HCV-infected patients is under way in our center. The study is investigating the prevalence of both rs12979860 and rs8099917 and their probable influences on the SVR rate among Iranian HCV-infected patients, and the results will be published soon.

**Table 1:** SNP genotyping results of the study group

	**Gender**		
	**Male** [No. (%)]	**Female** [No. (%)]	
**IL28**			
**CC**	24 (66.7%)	12 (33.3%)	36
**CT**	19 (70.4%)	8 (29.6%)	27
**TT**	3 (60%)	2 (40%)	5
**Total**	46	22	68

**Table 2:** HCV genotypes of the study group according to SNP genotyping

	**HCV Genotype**						**Total**
	**1**	**3**	**non-typable**	**3a and 1a**	**2**	**4**	
**IL28B**							
**CC**	21	9	2	1	1	0	34
**CT**	21	3	0	1	0	1	26
**TT**	2	1	0	0	0	0	3
**Total**	44	13	2	2	1	1	63
